# *Gnidia glauca*- and *Plumbago zeylanica*-Mediated Synthesis of Novel Copper Nanoparticles as Promising Antidiabetic Agents

**DOI:** 10.1155/2019/9080279

**Published:** 2019-02-11

**Authors:** Dhiraj A. Jamdade, Dishantsingh Rajpali, Komal A. Joshi, Rohini Kitture, Anuja S. Kulkarni, Vaishali S. Shinde, Jayesh Bellare, Kaushik R. Babiya, Sougata Ghosh

**Affiliations:** ^1^Department of Microbiology, Modern College of Arts, Science and Commerce, Ganeshkhind, Pune 411016, India; ^2^Institute of Bioinformatics and Biotechnology, Savitribai Phule Pune University, Pune 411007, India; ^3^Department of Applied Physics, Defense Institute of Advanced Technology, Girinagar, Pune 411025, India; ^4^Department of Chemistry, Savitribai Phule Pune University, Pune-411007, India; ^5^Department of Chemical Engineering, Indian Institute of Technology, Bombay, Powai, Mumbai 400076, India; ^6^Department of Microbiology, School of Science, RK University, Kasturbadham, Rajkot 360020, India

## Abstract

Rapid, eco-friendly, and cost-effective one-pot synthesis of copper nanoparticles is reported here using medicinal plants like *Gnidia glauca* and *Plumbago zeylanica*. Aqueous extracts of flower, leaf, and stem of *G. glauca* and leaves of *P. zeylanica* were prepared which could effectively reduce Cu^2+^ ions to CuNPs within 5 h at 100°C which were further characterized using UV-visible spectroscopy, field emission scanning electron microscopy, high-resolution transmission electron microscopy, energy dispersive spectroscopy, dynamic light scattering, X-ray diffraction, and Fourier-transform infrared spectroscopy. Further, the CuNPs were checked for antidiabetic activity using porcine pancreatic *α*-amylase and *α*-glucosidase inhibition followed by evaluation of mechanism using circular dichroism spectroscopy. CuNPs were found to be predominantly spherical in nature with a diameter ranging from 1 to 5 nm. The phenolics and flavonoids in the extracts might play a critical role in the synthesis and stabilization process. Significant change in the peak at ∼1095 cm^−1^ corresponding to C-O-C bond in ether was observed. CuNPs could inhibit porcine pancreatic *α*-amylase up to 30% to 50%, while they exhibited a more significant inhibition of *α*-glucosidase from 70% to 88%. The mechanism of enzyme inhibition was attributed due to the conformational change owing to drastic alteration of secondary structure by CuNPs. This is the first study of its kind that provides a strong scientific rationale that phytogenic CuNPs synthesized using *G. glauca* and *P. zeylanica* can be considered to develop candidate antidiabetic nanomedicine.

## 1. Introduction

Nature has an infinite collection of medicinal plants which serve as repository of bioactive principles that are considered as complementary and alternative medicine. Combinatorial chemistry, nanotechnology, and cutting edge research on nutraceuticals have helped to expand the horizons beyond contemporary therapeutics [1, 2]. Interdisciplinary research has enabled to exploit the medicinal plants which are storehouses of diverse groups of phytochemicals for fabrication of novel nanomedicine with broad-spectrum therapeutic applications [2–5]. Numerous medicinal plants like *Dioscorea bulbifera*, *Dioscorea oppositifolia*, *Gloriosa superba*, *Barleria prionitis*, and *Litchi chinensis* are used for synthesis of gold, silver, platinum, and palladium nanoparticles with antimicrobial, antibiofilm, and anticancer activities [6–12]. However, there is a lacuna in the area of synthesis of copper nanoparticles (CuNPs) using medicinal plants. Hereby, synthesis of therapeutic CuNPs using medicinal plants has drawn considerable attention recently. Among various nanoparticles, CuNPs have gained wide applications in photothermal ablation, photoacoustic imaging, drug delivery, theranostics, electrical conductors, biochemical sensors, electrocatalysis, photocatalysis, and catalytic organic transformations [13, 14]. Although there are various physical and chemical routes for synthesis of CuNPs, the involvement of hazardous and toxic chemicals poses a threat to the environment and compromises with the biocompatibility [15]. Hence, there is a growing need to develop the green synthesis approach for fabrication of stable CuNPs with therapeutic significance.

*Gnidia glauca* is also known to be of utmost medicinal importance as it is used for treatment of cancers, burns, wounds, abdominal pain, snake bites, and sore throat. Similarly, leaves are applied to treat back ache, joint aches, contusions, and swellings [16, 17]. Another medicinal plant, *Plumbago zeylanica*, has exhibited carminative, anthelmintic, anti-inflammatory, antiplasmodial, antimicrobial, antifungal, antihyperglycemic, hypolipidaemic, and antiatherosclerotic activities [18]. Further, it is used in the treatment of piles, rheumatic pain, diarrhoea, dysmenorrheal, anemia, contusion of the extremities, leprosy, ulcers, and furunculous scabies [19]. It is rich in coumarins like seselin, 5-methoxyseselin, suberosin, xanthyletin, and xanthoxyletin [19]. Alkaloids, glycoside, reducing sugars, simple phenolics, tannins, lignin, saponins, and flavonoids are found in the leaves of *P. zeylanica* which are caustic, vesicant, and aphrodisiac [20]. Thus, from the above information, it is evident that both *G. glauca* and *P. zeylanica* can be used for synthesis of metal nanoparticles since they are treasure house of both reducing as well as capping agents. However, there are no reports of synthesis and therapeutic applications of CuNPs from *G. glauca* or *P. zeylanica*. Hereby, there is a huge scope to design novel routes for synthesis of therapeutically active CuNPs using both the plants.

Development of antidiabetic nanomedicine is one of the thrust areas of nanotechnology as it is estimated that, by 2030, diabetes mellitus-afflicted population will shoot up to 366 million. Type II diabetes mellitus (T2DM) is the most prevalent ailment both globally as well as in the Indian subcontinent. Hereby, there is a continuous need to develop and screen novel antidiabetic agents that can target elevated postprandial hyperglycemia [17]. Herein, we report for the first time on the fabrication of CuNPs using *G. glauca* and *P. zeylanica* followed by characterization and evaluation of antidiabetic and antioxidant activity.

## 2. Materials and Methods

### 2.1. Chemicals

*α*-Glucosidase and 4-nitrophenyl *α*-D-glucopyranoside were obtained from Sigma Aldrich, USA. DNSA (3,5-dinitrosalicylic acid) was obtained from SRL Pvt. Ltd. (Mumbai, India). Copper sulphate, dipotassium hydrogen phosphate (K_2_HPO_4_), potassium dihydrogen phosphate (KH_2_PO_4_), methanol, sodium potassium tartarate, and sodium hydroxide (NaOH) were procured from Qualigens, Mumbai, India. Porcine pancreatic *α*-amylase and sodium chloride (NaCl) were obtained from HiMedia Laboratories, Mumbai, India. Acarbose was obtained from Bayer Pharmaceuticals Pvt. Ltd. (Mumbai, India). All the chemicals and reagents procured were of AR grade.

### 2.2. Plant Material and Preparation of Extract

*G. glauca* flowers, leaves, and stems and *P. zeylanica* leaves were collected from the Western Ghats of Maharashtra and shade-dried for 2-3 days at room temperature. The dried plant materials were reduced to fine powder using an electric blender. *G. glauca* flower extract (GGFE), leaf extract (GGLE), and stem extract (GGSE) were prepared by adding 5 g of the powdered plant material in 100 mL distilled water in a 250 mL Erlenmeyer flask, followed by boiling at 100°C for 5 min. Similarly, *P. zeylanica* leaf extract (PZLE) was prepared. After filtering the extract through a Whatman No.1 filter paper, the filtrate was collected and stored at 4°C for further use [21].

### 2.3. Synthesis and Characterization of Copper Nanoparticles

Synthesis of CuNPs was initiated by addition of 5 ml of GGFE, GGLE, GGSE, and PZLE separately to 95 ml of 1 mM aqueous CuSO_4_·5H_2_O solution and incubated in darkness at 100°C. UV-visible spectra were recorded at regular intervals on a spectrophotometer (SpectraMax M5, Molecular Devices Corporation, Sunnyvale, CA) operated at a resolution of 1 nm; also, visible colour change was monitored to confirm the reduction of Cu^2+^ ions to CuNPs. Bioreduced CuNPs were characterized by employing a field emission scanning electron microscope (FESEM), high-resolution transmission electron microscope (HRTEM), energy dispersive spectroscopy (EDS), dynamic light scattering (DLS), X-ray diffraction (XRD), and Fourier-transform infrared spectroscopy (FTIR) as per our earlier reports [22].

## 3. Glycosidase Inhibitory Activity

### 3.1. Porcine Pancreatic Amylase Inhibition Assay

In order to study the antidiabetic activity of the bioreduced CuNPs, *α*-amylase inhibitory activity was checked using the chromogenic 3,5-dinitrosalicylic acid (DNSA) method as per our earlier report [17]. In short, 10 *µ*g/mL CuNPs and porcine pancreatic *α*-amylase (50 *µ*g mL^−1^) were mixed and incubated for 10 min at 37°C. 1% starch was used as substrate which was added thereafter. DNSA assay was used to estimate the reducing sugar by recording the absorbance at 540 nm. Inhibitory activity was calculated by using the following formula:

(1) $$\begin{array}{c} \%\text{ inhibition}=\frac{\left({\text{A}540}_{\text{control}}-{\text{A}540}_{\text{test}}\right)}{\left({\text{A}540}_{\text{control}}\right)}\times 100. \end{array}$$

### 3.2. *α*-Glucosidase Inhibition Assays

Inhibition of *α*-glucosidase activity in presence of CuNPs was checked by mixing 100 *µ*L of *α*-glucosidase (0.1 unit/mL) with 200 *µ*L of CuNPs (100 *µ*g/mL) followed by incubation for 1 h at 37°C [1]. 10 mM *p*-nitrophenyl-*α*-D-glucopyranoside in 100 mM phosphate buffer of pH 6.8 was added to initiate the enzyme activity which was incubated for 10 min at 37°C and eventually stopped by addition of 2 mL Na_2_CO_3_ (0.1 M). Absorbance of the *p*-nitrophenol released from *p*NPG was recorded at 420 nm and percentage of glucosidase inhibition was evaluated using the following formula:

(2) $$\begin{array}{c} \%\text{ inhibition}=\frac{\left({\text{A}420}_{\text{control}}-{\text{A}420}_{\text{test}}\right)}{\left({\text{A}420}_{\text{control}}\right)}\times 100. \end{array}$$

### 3.3. Circular Dichroism (CD) Spectrometry

CuNPs were incubated with porcine pancreatic *α*-amylase and *α*-glucosidase separately at 37°C followed to which CD spectra were recorded on a Jasco J-1500 spectropolarimeter at a scan speed of 40 nm/min with a response time of 1 s and a slit width of 1 nm, as reported earlier. The measurements were recorded in a range from 190 to 300 nm in a Quartz cell of 2 mm path length using a reaction mixture comprising 0.1 unit/mL of enzyme and CuNPs in phosphate-buffered saline [23].

## 4. Results

### 4.1. UV-Visible Spectroscopy

**Figure 1:**
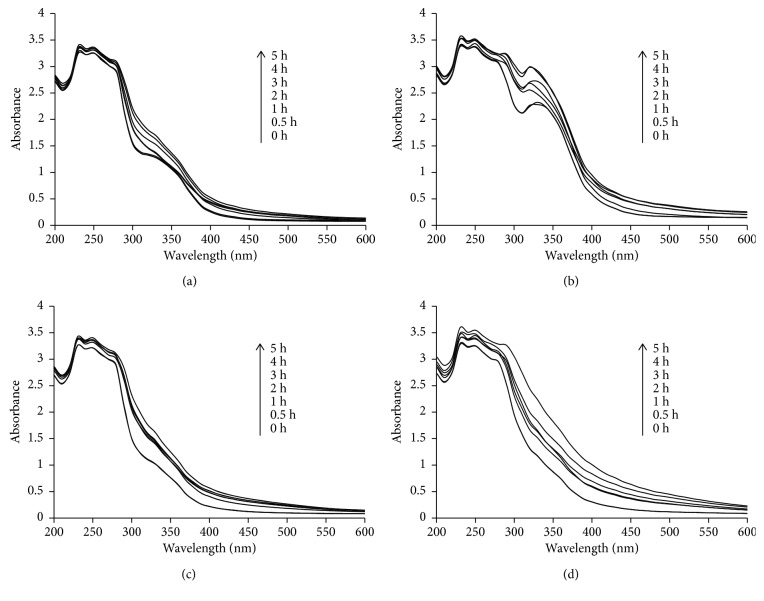
UV-visible spectra of CuNPs synthesized by plant extracts using 10^−3^ M aqueous CuSO_4_·5H_2_O solution in the dark at 100°C: (a) GGFE, (b) GGLE, (c) GGSE, and (d) PZLE.

Synthesis of CuNPs was confirmed by the visible colour change from pale blue to yellow and finally to dark brown. The intensity of the spectra increased up to 5 h followed by which no significant increase was noticed which indicated the completion of the bioreduction process in 5 h. A similar pattern in the enhancement of the spectral intensity was observed in all the cases where CuNPs were synthesized using GGFE, GGLE, GGSE, and PZLE at 100°C (Figure 1). This synthesis was found to be both rapid and efficient. The yield was found to be ∼62.66% from 100 mL reaction mixture.

### 4.2. HRTEM, EDS, DLS, and XRD Analysis

**Figure 2:**
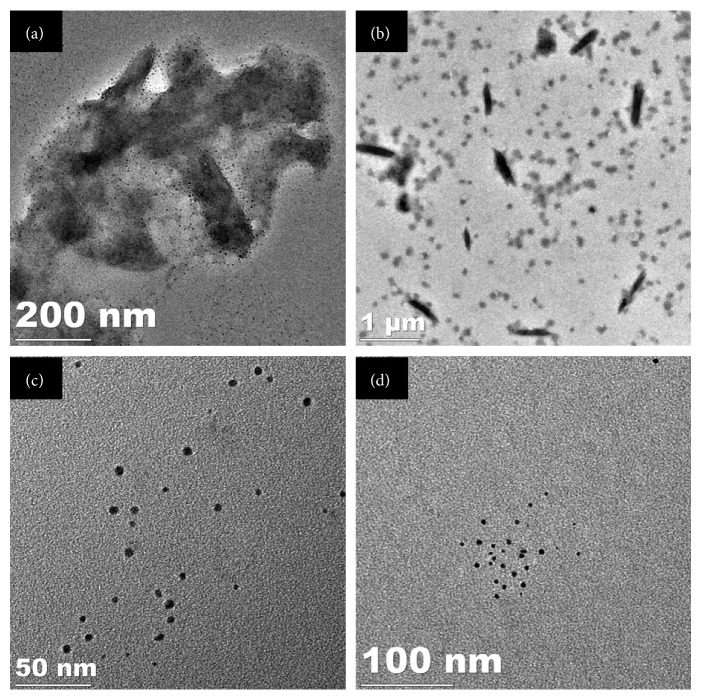
HRTEM micrographs of CuNPs synthesized by plant extracts using 10^−3^ M aqueous CuSO_4_·5H_2_O solution in the dark at 100°C: (a) GGFE, (b) GGLE, (c) GGSE, and (d) PZLE.

**Figure 3:**
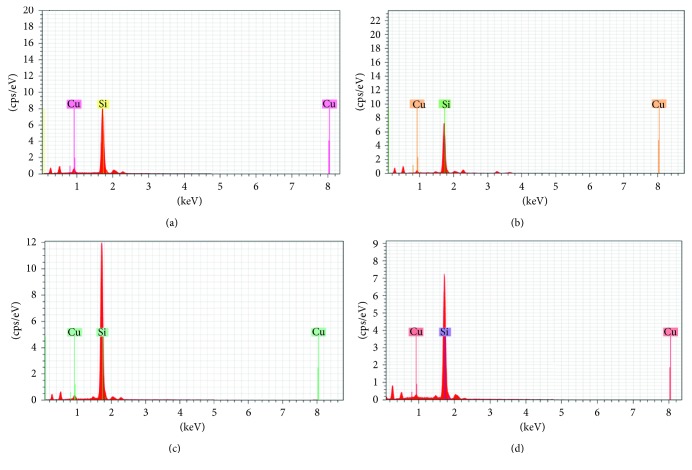
Spot EDS profile of CuNPs synthesized by plant extracts using 10^−3^ M aqueous CuSO_4_·5H_2_O solution in the dark at 100°C: (a) GGFE, (b) GGLE, (c) GGSE, and (d) PZLE.

**Figure 4:**
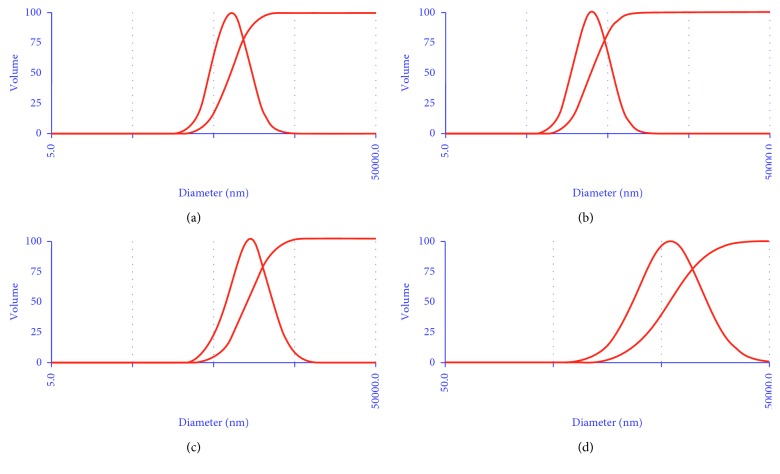
Particle size analysis using dynamic light scattering for CuNPs synthesized by plant extracts using 10^−3^ M aqueous CuSO_4_·5H_2_O solution in the dark at 100°C: (a) GGFE, (b) GGLE, (c) GGSE, and (d) PZLE.

Morphological features of the bioreduced CuNPs were evaluated using HRTEM analysis as FESEM failed to show high-resolution images since the CuNPs were very small in size (Figure S1). CuNPs were found to be embedded in the biological matrix which might play a critical role in synthesis and stabilization. It was observed that very small spherical nanoparticles were successfully fabricated which were found to be of 5 nm in size, when synthesized using GGFE (Figure 2(a)). However, the size of the CuNPs increased when synthesized using GGLE which was in a range between 70 and 93 nm, as found in case of the irregular brush border rods apart from the spherical ones (Figure 2(b)). In case of spherical CuNPs synthesized using GGSE, the particles were found to be monodispersed and discretely placed without any sign of aggregation or agglomeration indicating the high stability (Figure 2(c)). However, it is important to note that CuNPs synthesized using PZLE were found to be smaller with their size in a range between 1 and 5 nm (Figure 2(d)). EDS analysis revealed and confirmed that the nanostructures were composed of elemental copper (Figure 3). The high intensity peak of Si could be observed in EDS as particles were drop casted on silicon wafers and dried to do the analysis. Other peaks were due to sulphur and oxygen that might be an integral part of the biomolecule skeleton responsible for synthesis and capping of the CuNPs. Particle size analysis as evident from the DLS data can be closely correlated with the observed dimension recorded in HRTEM analysis (Figure 4). Increase in the particle diameter as observed in DLS might be attributed due to the close association of biological matrix around the CuNPs. XRD analysis is included in the supplementary information document (Figure S2). Although the nanoparticulate nature of the samples was observed through HRTEM, XRD data did not show distinct characteristic peaks of metallic CuNPs. There could be several reasons to this, including oxidation of copper when exposed to air during characterization and excess of plant extracts on the nanoparticles. Nevertheless, the EDS data confirmed the presence of CuNPs.

### 4.3. FTIR Analysis

**Figure 5:**
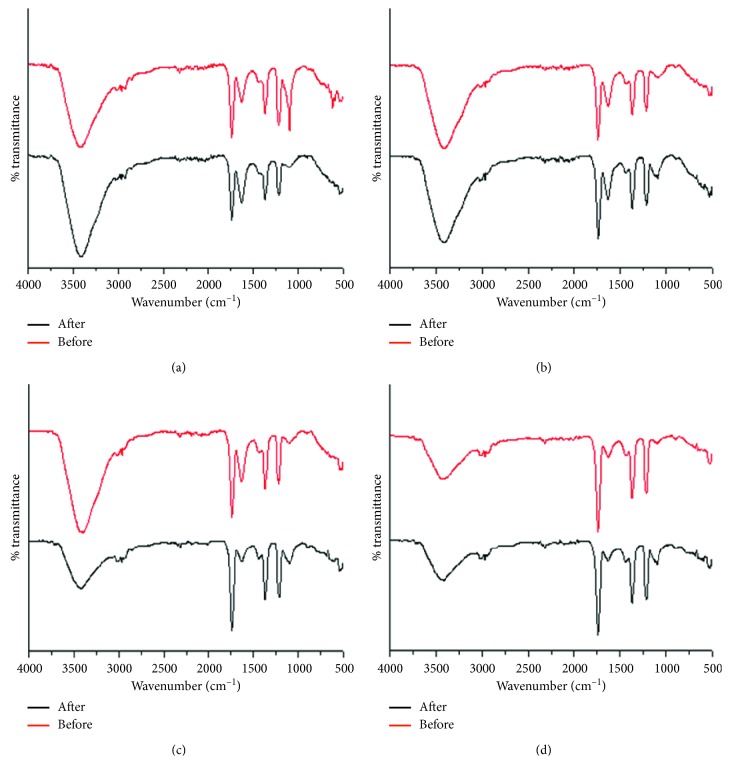
FTIR spectra of plant extracts before and after synthesis of CuNPs: (a) GGFE, (b) GGLE, (c) GGSE, and (d) PZLE.

The role of the extracts and the corresponding functional groups towards synthesis and stabilization of CuNPs was studied by recording the FTIR spectra of GGFE, GGLE, GGSE, and PZLE before and after synthesis of CuNPs (Figure 5). The plant extracts used for synthesis were recovered from the completed reaction mixtures and were independently added to KBr powder in order to record FTIR data. It is evident that all the four extracts showed similar characteristic peaks, before synthesis, indicating similar functional groups, irrespective of the plant part from which they are extracted. The variation in the intensity suggests variation in the concentration of the corresponding functional groups. All the four extracts exhibited strong characteristic peak at ∼3400–3420 cm^−1^ which is attributed to the hydroxyl group in alcoholic and phenolic compounds. However, no significant change was observed in the peaks after synthesis of CuNPs. The other significant and predominant peaks which were noticed before synthesis were not much altered after synthesis. The peaks at ∼1215 and ∼1624, 1365–1370, and ∼1740 cm^−1^ can be attributed to the unassigned amide mode, CH_3_ bend, and stretching of C=O bond, respectively. Remarkable change was observed in the peak at ∼1095 cm^−1^, which corresponds to C-O-C bond in ether. In case of GGFE, this peak was diminished significantly, while in rest of the extracts, there was notable change in the peak, after synthesis. This suggests that C-O-C bond is utilized during conversion of the Cu^2+^ to CuNPs. Moreover, minor change was also observed in the amide bond intensity, located at ∼1624 in case of GGSE, indicating its role in CuNPs. No prominent change in the rest of the peaks indicates that the corresponding functional groups help in stabilizing the as synthesized CuNPs.

### 4.4. Porcine Pancreatic *α*-Amylase Inhibition Assay

**Figure 6:**
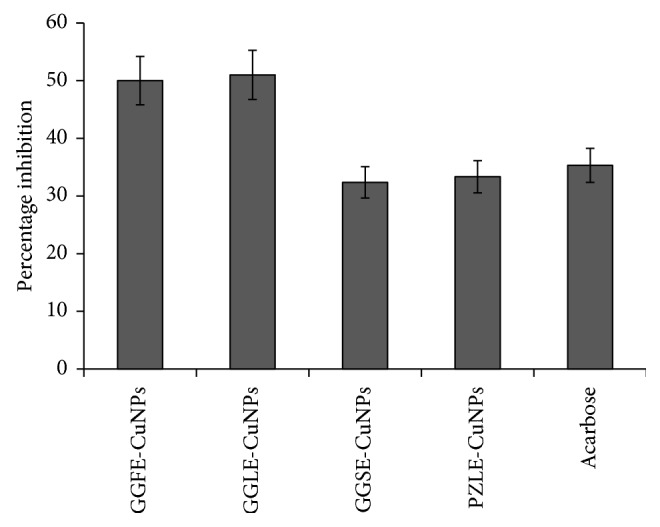
Porcine pancreatic *α*-amylase inhibition by CuNPs synthesized by different plant extracts: (a) GGFE, (b) GGLE, (c) GGSE, and (d) PZLE.

Bioreduced CuNPs showed promising inhibition against porcine pancreatic *α*-amylase (Figure 6). Among the various tested CuNPs samples, GGLE synthesized CuNPs showed the highest inhibition of porcine pancreatic *α*-amylase up to 50.99 ± 4.27% followed by GGFE-CuNPs that showed 50.01 ± 4.19%. CuNPs synthesized using GGSE (32.36 ± 2.71%) and PZLE (33.34 ± 2.79%) showed an inhibition equivalent to the standard drug acarbose (35.30 ± 2.95%).

### 4.5. *α*-Glucosidase Inhibition Assay

**Figure 7:**
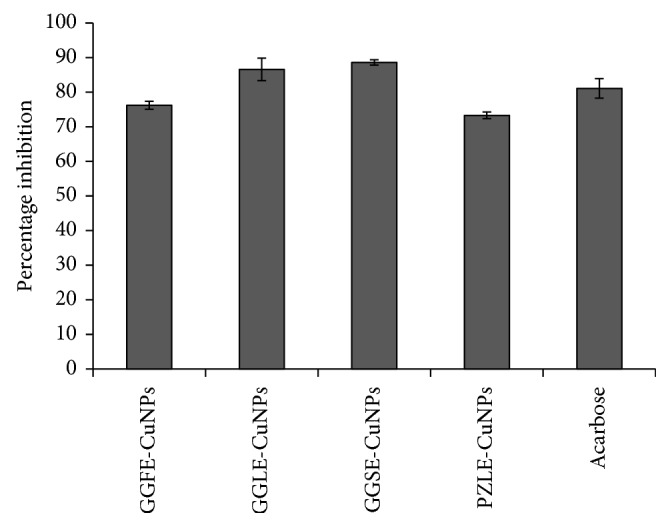
*α*-Glucosidase inhibition by CuNPs synthesized by different plant extracts: (a) GGFE, (b) GGLE, (c) GGSE, and (d) PZLE.

Among different CuNPs synthesized by 4 plant extracts, GGSE-synthesized CuNPs exhibited highest *α*-glucosidase inhibition up to 88.60 ± 0.78% followed by GGLE-synthesized CuNPs (86.58 ± 3.26%). Standard drug acarbose also showed similar inhibitory potential (81.09 ± 2.82%). CuNPs synthsized by GGFE and PZLE showed comparatively lower inhibitory potential up to 76.20 ± 1.14% and 73.29 ± 0.96%, respectively (Figure 7).

### 4.6. Circular Dichroism Analysis

**Figure 8:**
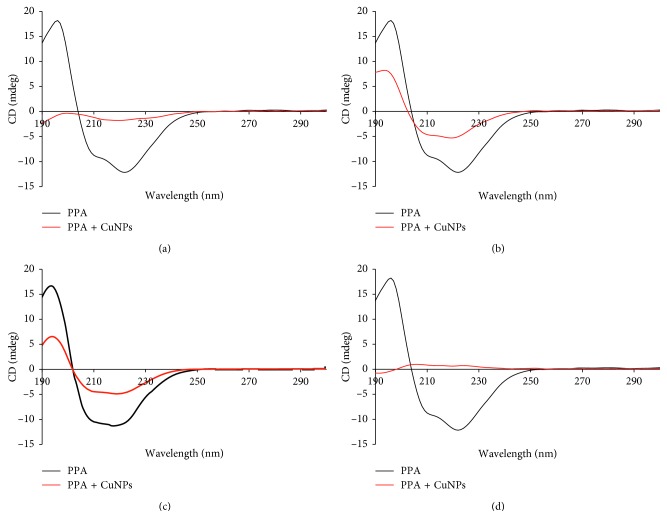
CD spectra of porcine pancreatic *α*-amylase inhibited by CuNPs synthesized by different plant extracts: (a) GGFE, (b) GGLE, (c) GGSE, and (d) PZLE.

**Figure 9:**
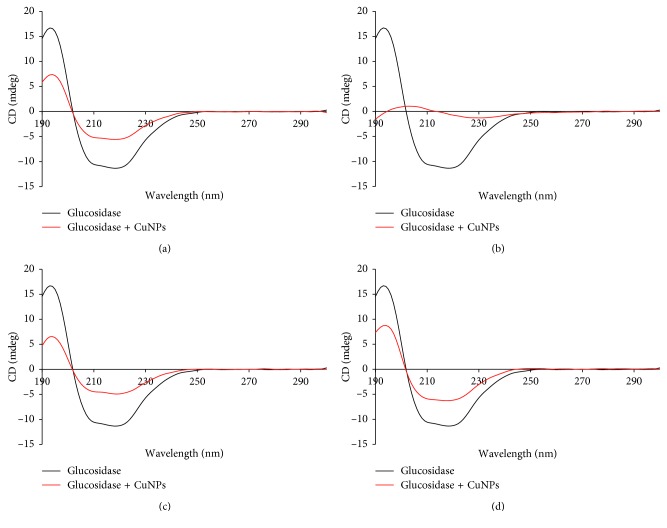
CD spectra of *α*-glucosidase inhibited by CuNPs synthesized by different plant extracts: (a) GGFE, (b) GGLE, (c) GGSE, and (d) PZLE.

Circular dichroism spectra confirmed the structural and conformational alteration in the enzymes in presence of CuNPs (Figures 8 and 9). Circular dichroism (CD) spectroscopy revealed the nature of interaction of porcine pancreatic *α*-amylase and *α*-glucosidase with CuNPs. As indicated by CD spectroscopy, the secondary structure of the enzymes was seen to be altered in presence of the CuNPs. Commonly, *α*-helical content of an enzyme shows two characteristic minima at 208 and 222 nm which were further compared after inhibition with CuNPs. The variation at 208 nm in the presence of CuNPs when compared with the control enzymes provided strong evidence that the interaction of CuNPs with the *α*-helix of both the enzymes resulted in a conformational change in the secondary structure of the enzymes.

## 5. Discussion

Metal nanoparticles have got wide applications in optoelectronics, semiconductors, sensors, and biomedical applications as well. Medicinal plants are widely explored to synthesize metal nanoparticles. In this study, we found that *G. glauca* and *P. zeylanica* have tremendous potential to synthesize and stabilize metallic CuNPs. In our previous studies, we have reported *G. glauca* flower-, leaf-, and stem-mediated synthesis of AuNPs and AgNPs [24–26]. However, there are no reports till date on their potential to synthesize bioactive CuNPs. Hereby, we have used three parts of *G. glauca*. Similarly, earlier, we could find that only *P. zeylanica* leaf can synthesize AuNPs, AgNPs, and bimetallic nanoparticles most effectively. But, till date, there are no reports of synthesis of CuNPs using *P. zeylanica* leaf extract [10]. In our present study, synthesis of CuNPs was found to be rapid and efficient which is well in agreement with our previous reports where AuNPs and AgNPs were synthesized using the aforementioned plants. The parts of the plants used in this study are reported to contain coumarins like seselin, 5-methoxyseselin, suberosin, xanthyletin, and xanthoxyletin apart from alkaloids, glycoside, reducing sugars, simple phenolics, tannins, lignin, saponins, and flavonoids which have a high potential to synthesize and stabilize nanoparticles [16, 17, 19]. Absorption bands of CuNPs are in the range between 550 and 600 nm. However, in our phytogenic approach, no sharp peak attributed to the surface plasmon resonance was observed which is well established by the earlier reports where similar observations were made for CuNPs coated with biomolecules [27]. Intensity of the UV-visible spectra progressively increases similar to the synthesis of CuNPs by hydroxyl ion-assisted alcohol reduction [28]. It can be rationalized by the earlier observations where freshly synthesized CuNPs (size, <5 nm diameter) at lower copper ion concentration demonstrated a featureless Mie scattering profile without the appearance of an apparent surface plasmon band which is in close agreement with our observation in the present study. This featureless broad peak may be attributed to the small size of bioreduced CuNPs [29]. Likewise, CuNPs synthesized using L-ascorbic acid were found to be less than 4 nm in diameter that exhibited a broadened peak and featureless absorbance, which increased monotonically towards higher energies. In our study as well, the bioreduced CuNPs did not show a plasmon peak at around 570 nm but rather displayed a broadened peak indicating the presence of a very small dimension of CuNPs which can be rationalized by the presence of ascorbic acid in the plant extracts that can lead to efficient reduction of Cu^2+^ to Cu^0^ and further more effective capping capacity [30–33]. High temperature was found to be suitable for synthesis of CuNPs which was evident from the visible colour change. Enhancement of the rate of synthesis of metal nanoparticles with rise in temperature is in close correlation with previous reports where the rate of synthesis of AgNPs using the *Lippia citriodora* leaf aqueous extract could be enhanced by increasing the temperature from 25°C to 95°C resulting in average particle size of 15–30 nm [34]. It is important to note that temperature plays a very critical role in the size and shape of the synthesized nanoparticles. Owing to the higher rate of reduction at higher temperatures, the copper ions could be consumed mainly on the formation of nuclei, whereas the secondary reduction process which takes place on the surface of the preformed nuclei might be hindered. This phenomenon is well documented during synthesis of AgNPs and AuNPs using *Lippia citriodora* and lemon grass, respectively [34, 35]. The rate of synthesis of AgNPs using aqueous extract of the leaves of *Mimosa pudica* could be effectively enhanced by heating the reaction mixture from ambient (29 ± 3°C) to 70°C. Moreover, increase in the reaction temperature evidently led to the synthesis of larger quantities of nanoparticles and, simultaneously, reduction in the size of the nanoparticles [36].

Synthesis and stabilization of the CuNPs by the both the plants may be attributed due to their rich phytochemistry. *G. glauca* is reported to have potent antioxidant activity owing to its high phenolic and flavonoid content [37]. Although mechanisms for synthesis of phytogenic nanoparticles are under exhaustive research, it is difficult to generalize a single factor for reduction of metal ions to their respective nanoparticles. Thus, in the light of previous reports, it may be hypothesized that multiple factors underlying the rapid synthesis of CuNPs in the present study may include reducing sugars (aldoses) and ketones, biomolecules with functional groups like–C–O–C, –C–O–, –C=C–, and–C=O–, derived from several heterocyclics, polyol components, flavonoids, and hydroxyls in the terpenoids. Further, peptides may also play a dual role for simultaneous reduction and capping. Earlier reports also rationalize the probability that quasi-spherical-shaped nanoparticles within a range between 10 and 30 nm may be synthesized by reductants like oxalic acid and aldehyde groups present in the plant extract [38]. In our previous studies, we have demonstrated that *P. zeylanica* has high concentration of phenolics, flavonoids, reducing sugar, citric acid, and plumbagin which might play a significant role in the process of bioreduction and capping [10]. FTIR analysis strongly rationalizes the interrelationship and interdependence of the phytochemical diversity and its role towards reduction and capping.

The phytogenic CuNPs could efficiently inhibit both *α*-amylase and *α*-glucosidase, which are key enzymes of carbohydrate metabolism. The variation in the extent of inhibition may be attributed due to the variation in size and shape. This fact may be evident from the observation where GGLE-synthesized CuNPs showed the highest inhibition of porcine pancreatic *α*-amylase while GGSE-synthesized CuNPs exhibited highest *α*-glucosidase inhibition. Type II diabetes mellitus (T2DM) is associated with postprandial hyperglycemia which can be effectively controlled using *α*-amylase and *α*-glucosidase inhibitors. However, the adverse effects associated with the available drugs like biguanides, thiazolidinediones, sulphonylureas, meglitinides, and *α*-glucosidase inhibitors in addition to insulin includes hepatotoxicity, abdominal pain, flatulence, diarrhoea, and hypoglycaemia [17, 39–42]. In this study, we found that, in the presence of CuNPs, the structural and/or conformational change in both *α*-amylase and *α*-glucosidase may be the most predominant mechanism of inhibition. Hereby, these phytogenic CuNPs may serve as novel complementary and alternative antidiabetic nanomedicine for the effective treatment and management of T2DM. Our findings are in close agreement with the previous reports where CuNPs and associated copper complexes were proved to be antidiabetic in nature. Recently, our findings about CuNPs, synthesized by *D. bulbifera* tuber extract, *B. prionitis* leaf extract, *L. chinensis* peel extract, and *P. orientalis* leaf extract, have shown spectacular success towards considering phytogenic CuNPs as potential candidate for designing antidiabetic nanomedicine [43]. Similarly, *Calotropis procera* L. latex is reported to produce highly biocompatible CuNPs which did not show any toxicity even at a concentration as high as 120 *µ*M which substantiates the biocompatibility of phytogenic CuNPs [44]. The yield of phytogenic CuNPs (62.66%) was found to be higher as compared to the chemical synthesis involving micelles of dodecylamine (yield 49%) while lower compared to CuNPs obtained with Triton X-100 (yield 99%) [45].

## 6. Conclusion

*G. glauca*- and *P. zeylanica*-mediated synthesis of CuNPs may prove to be a novel, rapid, and efficient route to fabricate spherical nanoparicles of smaller dimensions. The extracts rich in diverse phytochemicals not only bioreduce but also stabilize the nanoparticles as well. Elevated temperature was found to be suitable for synthesis. Phenolics and flavonoids might play a key role in the synthesis process. CuNPs could effectively inhibit *α*-amylase and *α*-glucosidase. The mechanism of enzyme inhibition was established to be alteration of the secondary structures in the enzyme leading to conformational change. In view of the background, it can be concluded that phytogenic CuNPs reported herein may lead to development of environmentally benign route for rational designing of safe and effective antidiabetic nanomedicine.

## Data Availability

The data used to support the findings of this study are available from the corresponding author upon request.

## Supplementary Materials

Supplementary Materials Figure S1: the FESEM micrographs of CuNPs synthesized by plant extracts using 10–3 M aqueous CuSO_4_·5H_2_O solution in the dark at 100°C. Figure S2: the X-ray diffraction profile of CuNPs.
