# Supplementary material for: Gnidia glauca- and Plumbago zeylanica-Mediated Synthesis of Novel Copper Nanoparticles as Promising Antidiabetic Agents
Source: Adv Pharmacol Sci. 2019 Feb 11;2019:9080279. doi: 10.1155/2019/9080279 (PMC6388358; doi:10.1155/2019/9080279)
Supplement: Supplementary Materials — Figure S1: the FESEM micrographs of CuNPs synthesized by plant extracts using 10–3 M aqueous CuSO4·5H2O solution in the dark at 100°C. Figure S2: the X-ray diffraction profile of CuNPs. [file 9080279.f1.pdf]

Supplementary Information

Gnidia glauca and *Plumbago zeylanica* mediated synthesis of novel copper nanoparticles as promising antidiabetic agents

Dhiraj A. Jamdade,[1] Dishantsingh Rajpali,[1] Komal A. Joshi,[2] Rohini Kitture,[3] Anuja S. Kulkarni,[4] Vaishali S. Shinde,[4] Jayesh Bellare,[5] Kaushik R. Babiya,[6] Sougata Ghosh[6*]

[1]*Department of Microbiology, Modern College of Arts, Science and Commerce, Ganeshkhind, Pune-411016, India;*
[2]*Institute of Bioinformatics and Biotechnology, Savitribai Phule Pune University, Pune-411007, India;*
[3]*Department of Applied Physics, Defense Institute of Advanced Technology, Girinagar, Pune-411025, India;*
[4]*Department of Chemistry, Savitribai Phule Pune University, Pune-411007, India;*
[5]*Department of Chemical Engineering, Indian Institute of Technology, Bombay, Powai, Mumbai-400076, India;*
[6]*Department of Microbiology, School of Science, RK University, Kasturbadham, Rajkot – 360020, India.*

directoribb@.unipune.ac.in

**List of figures**	**Page(s)**
FIGURE S1: FESEM micrographs of CuNPs synthesized by plant extracts using 10^{-3} M aqueous $CuSO_4.5H_2O$ solution in the dark at 100°C. A) GGFE; B) GGLE; C) GGSE and D) PZLE.	S2
FIGURE S2: Representative X-ray diffraction profile of CuNPs synthesized by plant extracts using 10^{-3} M aqueous $CuSO_4.5H_2O$ solution in the dark at 100°C. (Inset represent the extracts with which CuNPs are synthesized).	S3

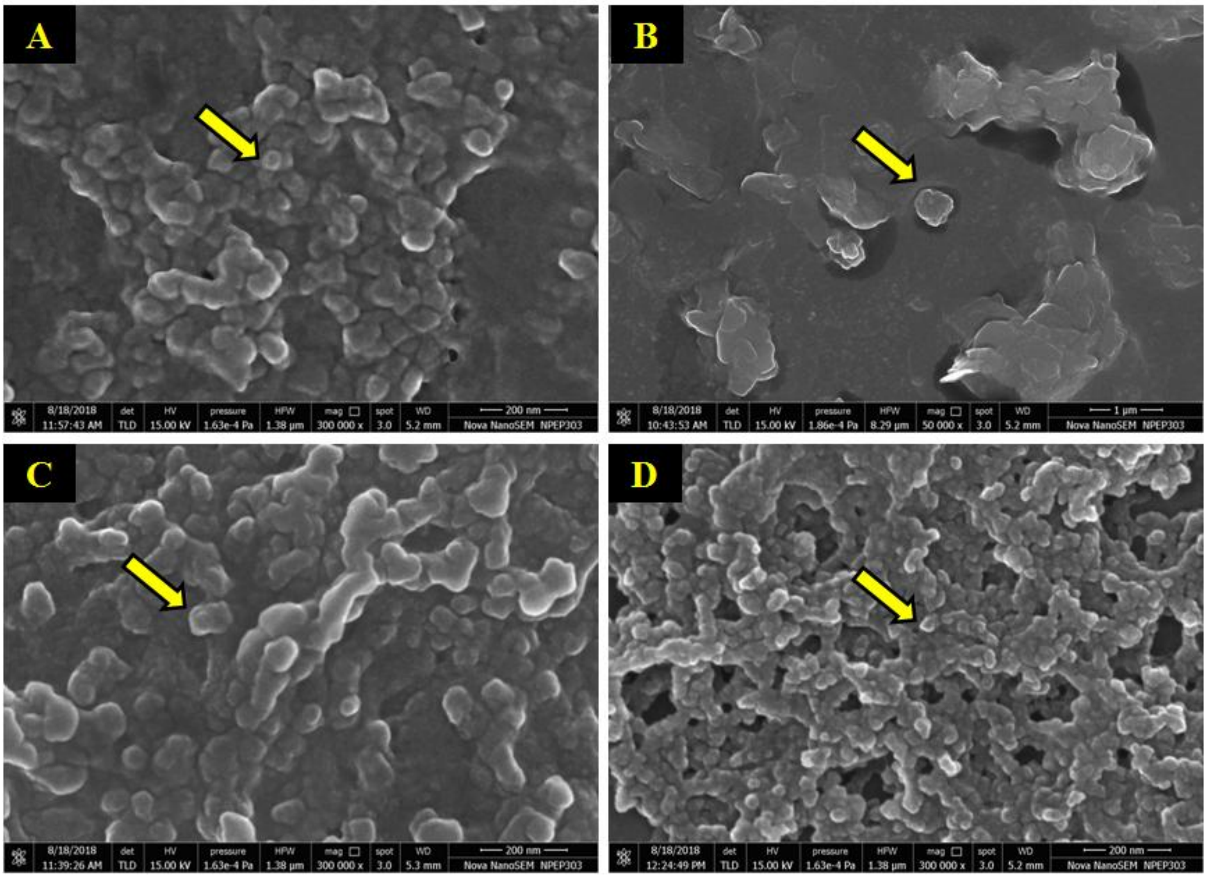

FIGURE S1: FESEM micrographs of CuNPs synthesized by plant extracts using 10^{-3} M aqueous $CuSO_4.5H_2O$ solution in the dark at 100°C. A) GGFE; B) GGLE; C) GGSE and D) PZLE.

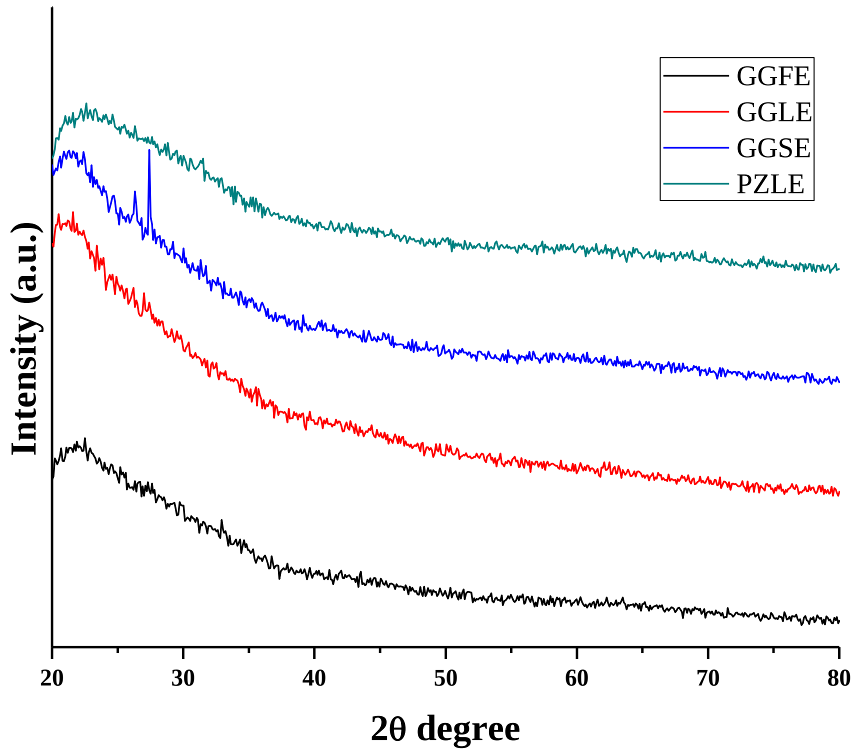

FIGURE S2: Representative X-ray diffraction profile of CuNPs synthesized by plant extracts using 10^{-3} M aqueous $CuSO_4.5H_2O$ solution in the dark at 100°C. (Inset represents the extracts with which CuNPs are synthesized).
